# Mitochondrial Protein Profile in Mice with Low or Excessive Selenium Diets

**DOI:** 10.3390/ijms17071137

**Published:** 2016-07-15

**Authors:** Lianmei Hu, Congcong Wang, Qin Zhang, Hao Yan, Ying Li, Jiaqiang Pan, Zhaoxin Tang

**Affiliations:** 1 College of Veterinary Medicine, South China Agricultural University, Guangzhou 510642, China; hulianmei@scau.edu.cn (L.H.); wcongcong321@126.com (C.W.); sunshine@scau.edu.cn (Q.Z.); yanhaoin1989@163.com (H.Y.); lying@scau.edu.cn (Y.L.); panjq@scau.edu.cn (J.P.); 2 Guangdong Provincial Key Laboratory of Prevention and Control for Severe Clinical Animal Diseases, Guangzhou 510642, China

**Keywords:** dietary selenium, mouse, hepatic mitochondrial proteomics, 2-dimensional electrophoresis, mass spectrometry

## Abstract

Dietary selenium putatively prevents oxidative damage, whereas excessive selenium may lead to animal disorder. In this study, we investigated the effects of low and excessive levels of dietary selenium on oxidative stress and mitochondrial proteins in mouse liver. Six to eight week old mice were fed a diet with low, excessive, or moderate (control) levels of selenium (sodium selenite). The selenium concentration and oxidative stress-related parameters in hepatic mitochondria were evaluated. Two-dimensional electrophoresis and mass spectrometry were applied to identify the differentially-expressed proteins associated with dietary selenium. The selenium content of the livers in mice with the low selenium diet was significantly lower than that of the control, while that of mice fed excessive levels was significantly higher. In both groups oxidative stress in hepatic mitochondria was found; accompanied by lower superoxide dismutase (SOD) and glutathione peroxidase (GPX) levels and higher malondialdehyde (MDA) content, compared with the control group. Furthermore, ten proteins in the hepatic mitochondria of the selenium-low or -excessive groups with more than two-fold differences in abundance compared with the control group were identified. The differentially-expressed proteins in hepatic mitochondria may be associated with dietary (low or excessive) selenium-induced oxidative stress.

## 1. Introduction

Selenium (Se) is an essential non-metal trace element in the mammalian diet. The functional role of selenium in regulating metabolic processes is largely attributed to its incorporation into selenoproteins [1]. For instance, glutathione peroxidases are one group of important selenoenzymes that participate in the redox regulation of a great number of biological processes implicated in cell life and death [2]. Dietary selenium improves various health conditions, and selenium deficiency has been linked to animal and human disorders [3]. In contrast, excessive selenium intake may lead to cytotoxicity, genotoxicity, and carcinogenesis [4,5]. The mechanism underlying these effects may be associated with excess accumulation of reactive oxygen species (ROS) and oxidative stress [5,6,7].

The metabolism and transport of selenium in the human body is primarily mediated by the liver [8]. A recent report showed that high level of selenium (sodium selenite) impairs hepatic insulin sensitivity [9]. In contrast, there is evidence that selenium protects against liver injury via antioxidant effects [10,11,12,13]. Shi et al. showed that, in duckling liver, selenium (sodium selenite) supplementation protects against hepatic mitochondrial antioxidant dysfunction induced by aflatoxin B_1_, and selenium is associated with attenuated mitochondrial permeability transition injury and DNA damage [10]. Moreover, in copper-overloaded rats, oral administration of sodium selenite is protective against structural alterations in hepatic mitochondria [14]. Therefore, selenium appears to be crucial for the maintenance of normal hepatic mitochondrial function. Nevertheless, the influence of dietary selenium deficiency or excessive on hepatic mitochondrial proteins has not been clearly elucidated.

Proteomic analysis of liver mitochondria is a valuable method for exploring the molecular mechanisms of hepatic disorders. Differentiable patterns in protein levels have been clarified in rodent models of different liver diseases via two-dimensional electrophoresis and matrix-assisted laser desorption/ionization tandem time-of-flight (MALDI-TOF) or liquid chromatography mass spectrometry (LC-MS/MS) [15,16].

To provide insights into the regulatory role of selenium on the oxidative processes of hepatic mitochondria, in the present study we investigated the effects of low or excessive selenium (sodium selenite) diet on the hepatic mitochondria of mice. Specifically, we evaluated the oxidative stress in hepatic mitochondria associated with malondialdehyde (MDA), superoxide dismutase (SOD), and glutathione peroxidase (GPX). In addition, we identified mitochondrial proteins that were found at differentiable levels in these mice.

## 2. Results

### 2.1. Selenium Concentration in Liver

**Figure 1:**
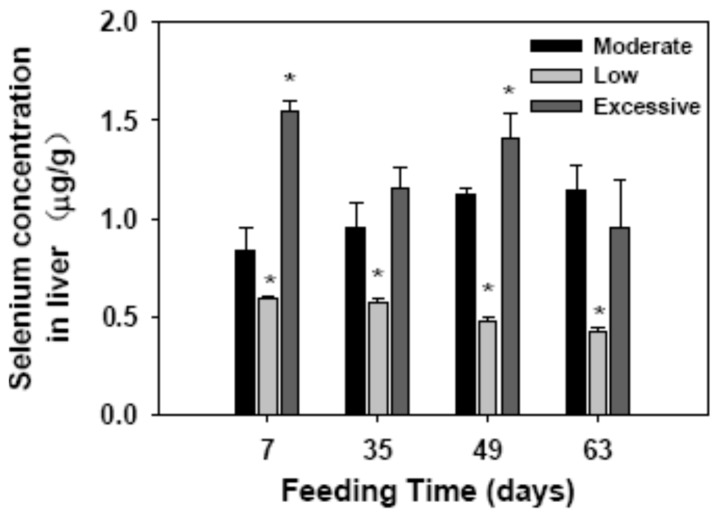
Selenium accumulation in the livers of mice fed different amounts of dietary selenium. Mice were fed low, excessive, or moderate (control) selenium diets, respectively. The liver selenium concentration was determined on the indicated day of consecutive feeding. Data were calculated from five mice in each group. * *p* < 0.05 compared with the control.

We investigated selenium content of the livers in the mice with low, excessive, or moderate (control) levels of selenium (sodium selenite) diets (Figure 1). In mice of the control group, the selenium content of the liver gradually increased during 63 days of the experimental period. In contrast, selenium in the livers of the mice feed low selenium diet gradually decreased and was significantly lower than that of the control group (*p* = 0.035 for all time points). The selenium content in the livers in the mice fed excessive amounts of dietary selenium was significantly higher than that of the control (*p* < 0.05).

### 2.2. Antioxidant Enzymes Activities and Lipid Perioxidation in Hepatic Mitochondria

**Figure 2:**
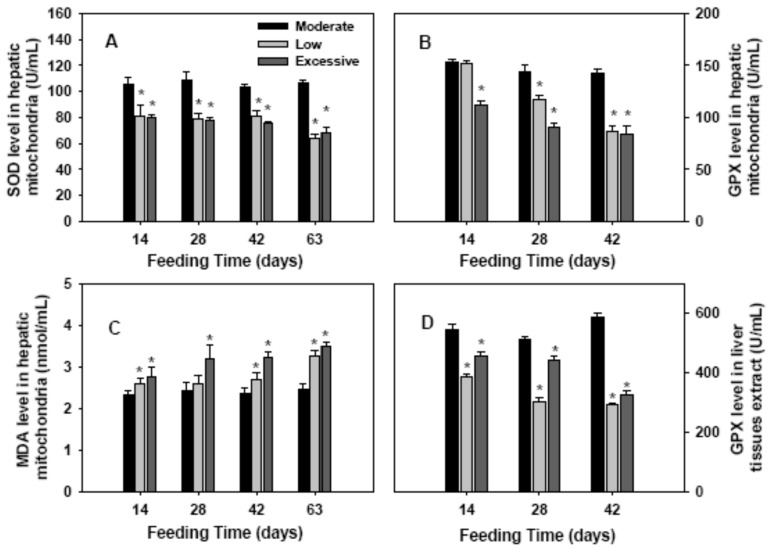
Antioxidant enzyme activities and lipid perioxidation in hepatic mitochondria or liver tissues extract. On the indicated day of consecutive feeding, the activities of superoxide dismutase (SOD) (**A**); glutathione peroxidase (GPX) (**B**); the level of malondialdehyde (MDA) (**C**) in hepatic mitochondria, and the GPX activity (**D**) in liver tissues extract. Data were calculated from five mice in each group. * *p* < 0.05 compared with the control group.

To evaluate the redox status in the hepatic mitochondria of the mice with different amount of selenium diets, SOD, GPX, and MDA levels of hepatic mitochondria were measured (Figure 2). On days 14, 28, 42, and 63, the SOD level in hepatic mitochondria was greatly lower in low and excessive selenium groups, compared with the control (*p* < 0.05; Figure 2A). Moreover, the GPX level was significantly lower in mice with low amounts of selenium diet since 28 days following feeding (*p* < 0.05), and the GPX level was dramatically lower in mice fed with excessive selenium since 14 days after feeding, compared with the control (*p* < 0.05; Figure 2B). In both the low and excessive selenium groups, the MDA levels were significantly higher than that in control group since 14 days following feeding (*p* < 0.05; Figure 2C). These data revealed that low and excessive selenium diets were associated with oxidative stress in hepatic mitochondria. In addition, the GPX activities in liver tissues extract in mice with low or excessive amounts of selenium diets were significantly lower than that in control mice (Figure 2D).

### 2.3. Differentially-Expressed Hepatic Mitochondria Proteins in Different Groups of Mice

**Figure 3:**
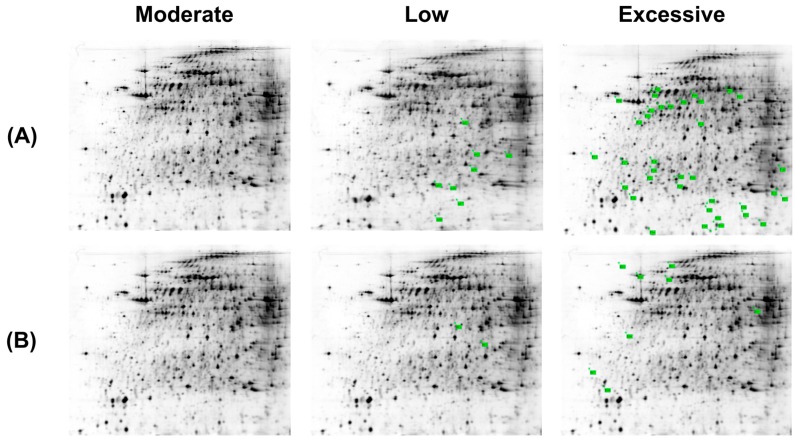
Two-dimensional electrophoresis of differentiable proteins in mitochondria. Mitochondria were extracted from the livers of mice with low, excessive or moderate levels of selenium diets. (**A**) Proteins that differed in amount by ≥1.5-fold increase, relative to the control, are indicated in green; and (**B**) proteins that differed in amount by ≥1.5-fold decrease, relative to the control, are indicated in green.

Considering that the above results suggested that dietary selenium may be associated with oxidative stress in hepatic mitochondria, we further compared the protein profiles of hepatic mitochondria in different groups of mice using two-dimensional electrophoresis (Figure 3). In low, excessive, and moderate (control) selenium groups, 243, 227, and 246 protein spots were identified, respectively, and the match rates were high among the three groups (98% between the deficient and control; 99% between the excessive and control).

Hepatic mitochondrial proteins that were higher or lower in amounts by 1.5-fold of the control group were considered to be differentially expressed. Based on this criterion, 10 differentially-expressed proteins (two downregulated and eight upregulated) were identified in the low selenium group, and in the excessive selenium group there were 42 differentially-expressed proteins (eight downregulated and thirty-four upregulated). Thus, dietary selenium was associated with a total of 52 differentially-expressed proteins.

Of the 52 differentially-expressed proteins, 10 proteins with more two-fold differences in abundance compared with the control group were subjected to MALDI-TOF or LC-MS/MS analysis (specifically, three downregulated and one upregulated in the low selenium group; and three downregulated and three upregulated in the excessive selenium group). Eight differentially-expressed proteins were identified (Table 1 and Table 2): ATP synthase subunit d (ATPsyn-d) in mitochondrial complex F0; ATP synthase subunit α (ATPsyn-α) in mitochondrial complex F1; 17β hydroxysteroid dehydrogenase (17β-HSD); elongation factor 2 (EF2); carbamoyl phosphate synthetase 1 (CPS1); glutathione peroxidase 1 (GPX1); heat shock protein 90 kDa (HSP90); subunit β of mitochondrial succinyl-CoA ligase (SUCLA2); and two unknown proteins.

In the mice fed a low selenium diet, the levels of ATPsyn-d in mitochondrial complex F0 and 17β-HSD were significantly higher, while that of EF2 was dramatically lower, compared with the control. In the mice with excessive selenium diet, the levels of ATPsyn-α and CPS1 were greatly higher, whereas the expression of GPX1, HSP90, and SUCLA2 were lower.

These findings suggest that dietary selenium is related to the differences in mitochondrial protein expression in mice liver, and these differences may be implicated in oxidative damage in hepatic mitochondria.

## 3. Discussion

In this study, we investigated the potential influence of low or excessive selenium diets on oxidative stress and hepatic mitochondria proteins in mice. Using two-dimensional electrophoresis, ten proteins in the hepatic mitochondria of the low or excessive selenium groups with more two-fold differences in abundance were identified.

In biological contexts, ROS are formed as a natural byproduct of normal oxygen metabolism. ROS play important roles in cell signaling and homeostasis. However, excessive accumulation of ROS usually causes oxidative damage of macromolecules, such as proteins and lipids. To protect cells from ROS damage, organisms possess very efficient enzymatic antioxidant systems, of which SOD catalyzes the dismutation of superoxide radical to H_2_O_2_ while glutathione peroxidases are enzymes that catalyze the reduction of H_2_O_2_ and hydroperoxides to H_2_O or alcohols [17]. In the present study, the SOD and GPX levels of hepatic mitochondria in mice fed either low or excessive amounts of selenium were significantly lower than that of the control mice within 28 days of the start of the experiment. Additionally, GPX activity in liver tissue extracts in mice with low or excessive selenium diets was lower than that in the control mice. In contrast, the MDA levels, a product of lipid peroxidation, in both groups were higher than that in the control group. These results indicated that low or excessive selenium diets resulted in decreased antioxidant activity and increased lipid peroxidation.

GPXs are one group of selenium-containing enzymes. Selenium influences the activities of GPXs and other antioxidant enzymes. Gan et al. reported that adequate selenium (sodium selenite) results in the increased GPX activities in the rat liver while intraperitoneal injection of 40 and 80 μg Se/kg/d dramatically decrease the activity in liver [18]. Krohn et al. also found that moderate dietary selenium (from lentil) supplementation (>0.16 mg/kg) is associated with antioxidant defense against arsenic-induced hepatic oxidative stress in mice [19]. It has also been reported that either deficient or excessive dietary selenium (sodium selenite) cause oxidative stress in the liver of the juvenile yellow catfish *Tachysurus* *fulvidraco* [20]. Therefore, adequate dietary selenium was required for the maintenance of antioxidant enzyme system and helped to prevent hepatic mitochondria oxidative stress. However, low or excessive selenium had an adverse effect on antioxidant activities. Furthermore, it was noted that the mice fed low or excessive selenium diets for 63 days had similar decreases in SOD and GPX activities and a similar increase in MDA level in liver.

By incubating HEK293 cells with selenium (sodium selenite), Touat-Hamici et al. detected a selective upregulation of several selenoproteins involved in antioxidant defense in response to oxidative stress [21]. In the present study, we investigated for the first time the differentially-expressed proteins in hepatic mitochondria of mice fed low or excessive levels of dietary selenium. We detected 10 differentially-expressed proteins (two downregulated and eight upregulated) in the hepatic mitochondria of mice with low selenium diet, relative to the control mice. Of these proteins, eukaryotic elongation factor 2 (eEF2) was significantly downregulated. eEF2 is involved in the elongation of mRNA translation, which promotes the GTP-dependent translocation of the nascent protein chain from the A-site to the P-site of the ribosome [22]. In hippocampal neurons, oxidative stress induces the hyperphosphorylation and ribosylation of EF2, leading to a decline in translational activity [23]. Therefore, the downregulation of EF2 in hepatic mitochondria observed in the present study might be associated with low selenium-induced oxidative stress. Furthermore, the level of 17β-hydroxysteroid dehydrogenase (17β-HSD) was also remarkably higher in the hepatic mitochondria of mice given the low selenium diet, compared with the control group. 17β-HSDs are enzymes involved in the metabolism of steroids and lipid [24]. The lack of dietary selenium may affect steroid metabolism in mice. Su et al. reported 17β-HSD13 as a pathogenic protein in the development of nonalcoholic fatty liver disease (NAFLD) [25]. Our results indicated that the low selenium diet altered the metabolism of steroids and lipid in mice liver. In addition, the level of ATPsyn-d in the mitochondrial complex F0, which is responsible for hydrogen ion (H^+^) transport during ATP synthesis [26], was significantly higher compared with the control.

In mice fed excessive amounts of selenium, the accumulation of GPX1, HSP90, and SUCLA2 were significantly downregulated compared with the control group. GPX1 is an important antioxidant enzyme that plays a crucial role in preventing excess accumulation of intracellular hydrogenperoxide, lipid hydroperoxides and other soluble hydroperoxides [27,28]. There is mounting evidence that GPX1 is involved in modulating cellular oxidative stress and redox-mediated response. Valencia et al. reported that the presence of the ovary prevents hepatic mitochondrial oxidative stress in young and aged female mice through glutathione peroxidase 1 [29]. Mai et al. found that genetic over-expression of GPX1 attenuates cocaine-induced renal toxicity via induction of anti-apoptotic factors [30]. In this study, the downregulated accumulation of GPX1 in mice fed an excessive selenium diet lowered the efficiency of ROS scavenging, which was associated with increased oxidative stress. In accordance with our results, Gan et al. reported that intraperitoneal injection of excessive selenium (sodium selenite) dramatically decreased GPX mRNA in liver of rat [18]. In addition, a recent study has showed that supranutritional selenium (sodium selenite) resulted in downregulation of selenoprotein genes in three immune organs of chicken [31]. Heat shock proteins (HSPs) are stress-responsive family of proteins, which are involved in posttranslational modifications, protein folding, and aggregation and disaggregation of proteins. Heat shock protein 90 (HSP90) is a ubiquitous molecular chaperone involved in conformational stabilization, maturation and activity of its client proteins [32]. In addition, HSP90 assists the degradation of oxidized proteins and thereby prevents oxidative stress in cells [33]. Under excessive selenium, the downregulated expression of HSP90 was not advantage for the degradation of oxidized proteins, which in turn aggravated oxidative damage. Succinyl-CoA ligase (SUCL) is a mitochondrial enzyme in the Krebs that converts succinyl-CoA to succinate and free CoA [34]. This enzyme is composed of two subunits, of which β-subunit is encoded by SUCLA2 or SUCLG2, depending on whether the substrate is ADP or GDP, respectively. In patients with severe hypotonia, deafness, and Leigh-like syndrome, mutations have been found in SUCLA2, which is associated with mtDNA depletion [35]. In the present study, lower levels of SUCLA2 in the hepatic mitochondria in mice feed excessive selenium diet, relative to the control, suggested impaired mitochondrial function. 

In addition to the downregulated expression of GPX1, HSP90, and SUCLA2, the expression of F1-ATPsyn-α and CPS1 were greatly upregulated in mice fed excessive amounts of selenium. F1-ATPase subunits are inner mitochondrial membrane enzymes implicated in liver disorders, such as primary biliary cirrhosis [36]. The increased level of F1-ATPsyn-α observed in our study may be explained by disturbed ATP synthesis in hepatic mitochondria due to excessive selenium englobement. CPS1 is an enzyme that serves as a crucial regulator of the urea cycle in the mitochondrial matrix and involved in the clearance of excess ammonia from the cell [37]. In the present study, the higher levels of CPS1 associated with excessive selenium feeding suggests an increase in urea cycle burden.

In summary, our present study identified several differentially-expressed mitochondrial proteins in the livers of mice fed either low or excessive selenium diets. These findings suggest that adequate selenium supplementation is important for the maintenance of ROS homeostasis and mitochondrial metabolism in cells. Either low-dose or overdose selenium may lead to oxidative stress in hepatic mitochondria and subsequently cause liver disorder. However, this study did not characterize the number, size, and function of mitochondria in mice with low and excessive levels of selenium diets, which possibly is associated with liver disorder. In addition, only one selenium species (sodium selenite) was applied in the present study. Other forms of selenium, such as selenate and selenomethionine, may have a differential impact on oxidative stress and protein profile in mitochondria.

## 4. Materials and Methods

### 4.1. Reagents

Sodium selenite (purity, 99.99%) was purchased from Jianglaibio (Shanghai, China). The low selenium diet was provided by Guangdong Medical Laboratory Animal Center (Guangzhou, China). Urea, thiourea, and phenylmethanesulfonyl fluoride (PMSF) were purchased from Amersham (Pittsburgh, PA, USA).

### 4.2. Establishing a Mouse Model

One hundred and fifty male BALB/C mice, 6–8 week old, each weighing ~20 g, were obtained from Guangdong Medical Laboratory Animal Center, China. Animals were housed at 22 ± 1 °C with constant humidity and a 12-h light/dark cycle. All the mice had free access to water prior to, and throughout, the experimental period. Mice were adapted to the housing conditions and normal diet containing 0.1 mg/kg selenium for one week prior to experiments.

The mice were randomly and equally apportioned into three groups. These groups were fed a diet deficient in selenium (0.045 mg/kg), excessive levels of selenium (0.78 mg/kg), or a moderate amount of selenium (0.13 mg/kg; the control), as reported previously [38].

Compositions of selenium-deficient diet were as follows: yeast extract (40 g/kg), sucrose (45.25 g/kg), soybean oil (5 g/kg), cellulose (5 g/kg), dl-methionine (0.4 g/kg), choline chloride (0.25 g/kg), mineral mix (AIN-93G-MX) (3.5 g/kg), Vitamin mix (AIN-93G-VM) (1 g/kg). Of the compositions, mineral mix includes anhydrous calcium carbonate (357 g/kg mix), potassium phosphate monobasic (196 g/kg mix), potassium citrate (70.78 g/kg mix), sodium chloride (74 g/kg mix), potassium sulfate (46.6 g/kg mix), magnesium oxide (24 g/kg mix), ferric citrate (6.06 g/kg mix), zinc carbonate (1.65 g/kg mix), manganese carbonate (0.63 g/kg mix), copper carbonate (0.3 g/kg mix), potassium iodide (0.01 g/kg mix), sodium selenite (0.01 g/kg mix), ammonium molybdate (0.01 g/kg mix), while the vitamin mix includes vitamin B1 (0.60 g/kg mix), vitamin B2 (0.60 g/kg mix), vitamin B3 (3.00 g/kg mix), vitamin B5 (1.60 g/kg mix), vitamin B6 (0.70 g/kg mix), vitamin B12 (2.50 g/kg mix), vitamin A (1.23 g/kg mix), vitamin D5 (1.00 g/kg mix), vitamin E (15.00 g/kg mix), vitamin K (1.50 g/kg mix), folic acid (0.20 g/kg mix), biotin (1.00 g/kg mix). The selenium content in selenium-deficient diet was 0.045 mg/kg by atomic fluorescence spectrophotometer. Control and selenium-excessive diets were attained by adding selenium to selenium-deficient diet to reach the levels of 0.13 and 0.78 mg/kg, respectively.

Animal studies have been approved by the Animal Ethical Committee of South China Agriculture University.

### 4.3. Determination of the Selenium Concentration in Liver

On 14, 28, 42, 63 days of consecutive feeding, five animals in each group were killed. Liver tissues were minced and 0.2 g of were used for determination of the selenium concentration. The selenium concentration in the livers was examined by atomic fluorescence spectrophotometer (AFS-9130, Beijing Titan Instruments, Beijing, China).

### 4.4. Sample Preparation and Biochemical Analyses

At 14, 28, 42, and 63 days after the start of the dietary selenium treatments, blood samples were collected from the orbital sinus of each mouse. Serum levels of alanine aminotransferase (ALT) and aspartate aminotransferase (AST) were measured. Animals were killed, and the liver tissues were removed and stored at −80 °C until used.

### 4.5. Isolation of Hepatic Mitochondria

On 14, 28, 42, 63 days of consecutive feeding, five animals in each group were killed. Hepatic mitochondria were isolated from liver tissues as described previously [39]. In brief, cells were homogenized in ice-cold isolation buffer supplemented with 10 mM KCl, 1.5 mM MgCl_2_, 20 mM pH 7.2 HEPES-KOH, 250 mM sucrose, 1 mM PMSF, 10 μg/mL leupeptin, 10 μg/mL pepstatin, 10 μg/mL aprotinin, and 1 mM dithiothreitol.

The samples were centrifuged at 750× *g* for 5 min at 4 °C, and the supernatant was collected and centrifuged at 10,000× *g* for 15 min at 4 °C. The pellet (mitochondrial fraction) was resuspended in isolation buffer, and the concentration of hepatic mitochondria was determined by Bradford assay. The MDA content and activities of SOD, and GPX in hepatic mitochondria were examined using specific kits in accordance with the manufacturer’s instructions (Nanjing Jiancheng Bioengineering Institute, Nanjing, Jiangsu, China).

### 4.6. Sample Preparation for Two-Dimensional Gel Electrophoresis

Mitochondrial samples were collected from five animals of each group on 63 days of consecutive feeding. Samples were lyzed in lysis buffer containing 20 mM Tris, 2 M thiourea, 7 M urea, 2% (*w*/*v*) Chaps cell extract buffer, and 1 mM PMSF. After sonication, samples were centrifuged at 12,000× *g* for 20 min at 4 °C. The supernatant was collected, and proteins were precipitated by incubating with acetone at −20 °C overnight. On the next day, samples were centrifuged at 12,000× *g* for 20 min at 4 °C and the supernatant was discarded. Proteins were dried by air and then stored at −80 °C until used.

### 4.7. Electrophoresis and Spot Detection

Before isoelectric focusing, samples were rehydrated in rehydration buffer stock containing 2 M thiourea, 7 M urea, and 4% (*w*/*v*) Chaps cell extract buffer. Isoelectric focusing was conducted using a 5-step protocol as follows: stp (speediness) 300 V for 30 min; stp 700 V for 30 min; stp 1500 V for 90 min; grd (linearity) 9000 V for 3 h; and stp 9000 V for 4 h, to accumulate 52,000 total volt-hours. Equilibrated immobilized pH gradient gel strips were then placed onto a 12.5% SDS-PAGE gel. Silver stained gel was scanned using a UMAX Powerlook 1100 scanner (Taibei, Taiwan). The number of spots in the individual gels was analyzed using a Image Master 2D platinum 5.0 software (GE, Pittsburgh, PA, USA).

### 4.8. Mass Spectrum Analyses

The spots of interests were excised from the 2-dimensional gel and were subjected to MALDI-TOF or LC-MS/MS analysis using an AutoFlex MALDI-TOF-TOF instrument (Bruker Dalton, Billerica, MA, USA). Data were searched in the matrix science Mascot search engine using the following parameter settings: type of search, MS/MSI on search; enzyme, trypsin; fixed modifications, carbamidomethyl; variable modifications, Gln->pyro-Glu (N-term Q), oxidation (M); mass values, monoisotopic; protein mass, unrestricted; peptide mass tolerance, ±100 ppm; fragment mass tolerance, ±0.5 Da; maximum missed cleavages, 1; instrument type, MALDI-TOF-TOF; number of queries, 50.

### 4.9. Statistical Analysis

Data are presented as mean ± standard error. Data were analyzed using SPSS 16.0 software (IBM SPSS, Armonk, NY, USA). Statistical significance was determined via one-way analysis of variance. *p* < 0.05 was recognized as significantly different.

## Figures and Tables

**Table 1:** Differentially-expressed proteins in hepatic mitochondria of mice fed a low selenium diet.

Spot	Description	Accession	Score	Mr	Sequence Coverage	pI	Expression *
A04	mCG116926	gi|148671376	80	35,753	24	7.72	↑
A05	ATP synthase, H^+^ transporting, mitochondrial F0 complex, subunit d	gi|159572410	76	15,929	37	5.16	↑
A07	Hydroxysteroid (17-β) dehydrogenase 4	gi|148677986	107	32,826	17	8.76	↑
B01	Eukaryotic elongation factor 2	gi|192989	137	30,212	30	6.2	↓

pI, isoelectric point; Mr, relative molecular mass. * the differences are compared with the adequate feeding group (Control group).

**Table 2:** Differentially-expressed proteins in hepatic mitochondria of mice fed an excessive selenium diet.

Spot	Description	Accession	Score	Mr	Sequence Coverage	pI	Expression *
E03	HSP90	gi|6755863	65	92,703	12	4.74	↓
E06	SUCLA2	gi|28175163	147	38,384	8	5.05	↓
F10	CPS1	gi|187466221	101	128,299	5	6.22	↑
F13	mCG121563	gi|148667830	208	158,788	5	6.59	↑
F26	GPX1	gi|84871986	137	22,544	27	6.74	↓
F27	ATP synthase, H^+^ transporting, mitochondrial F1 complex, α subunit	gi|148677503	203	30,708	35	8.98	↑

pI, isoelectric point; Mr, relative molecular mass. * the differences are compared with the adequate feeding group (control group). HSP90, heat shock protein 90 kDa; SUCLA2, subunit β of mitochondrial succinyl-CoA ligase; CPS1, carbamoyl phosphate synthetase 1; GPX1, glutathione peroxidase 1.

## Abbreviations

17β-HSD	17β hydroxysteroid dehydrogenase
ALT	alanine aminotransferase
AST	aspartate aminotransferase
ATPsyn-d	ATP synthase subunit d
ATPsyn-α	ATP synthase subunit α
CPS1	carbamoyl phosphate synthetase 1
EF2	elongation factor 2
GPX	glutathione peroxidase
HSP90	heat shock protein 90 kDa
LC-MS/MS	liquid chromatography mass spectrometry
MALDI-TOF	matrix-assisted laser desorption/ionization tandem time-of-flight
MDA	malondialdehyde
PMSF	phenylmethanesulfonyl fluoride
Se	Selenium
SOD	superoxide dismutase
SUCLA2	subunit β of mitochondrial succinyl-CoA ligase
